# The characteristics of upper airway edema in hereditary and acquired angioedema with C1‐inhibitor deficiency

**DOI:** 10.1002/clt2.12083

**Published:** 2021-12-07

**Authors:** Zsuzsanna Balla, Noémi Andrási, Zsófia Pólai, Beáta Visy, Ibolya Czaller, György Temesszentandrási, Dorottya Csuka, Lilian Varga, Henriette Farkas

**Affiliations:** ^1^ Department of Internal Medicine and Haematology Hungarian Angioedema Center of Reference and Excellence Semmelweis University Budapest Hungary; ^2^ School of PhD Studies Semmelweis University Budapest Hungary; ^3^ 2nd Department of Pediatrics Semmelweis University Budapest Hungary; ^4^ Heim Pál Children's Hospital Budapest Hungary; ^5^ Department of Pulmonology Semmelweis University Budapest Hungary; ^6^ Hospital of the Hospitaller Brothers of Saint John of God Budapest Hungary; ^7^ Research Laboratory Department of Internal Medicine and Haematology Semmelweis University Budapest Hungary; ^8^ MTA‐SE Research Group of Immunology and Hematology Hungarian Academy of Sciences and Semmelweis University Budapest Hungary

**Keywords:** acquired angioedema, C1‐inhibitor deficiency, hereditary angioedema, laryngeal edema, upper airway edema, C1‐inhibitor‐Mangel, erbliches angioödem, erworbenes angioödem, kehlkopfödem, ödem der oberen atemwege

## Abstract

**Background:**

Angioedemas localized in the upper airway are potentially life threatening, and without proper treatment, they may lead to death by suffocation. Upper airway edemas (UAE) in bradykinin‐mediated angioedemas can even be the first symptoms of the disease.

**Methods:**

Our survey was performed with a retrospective long‐term follow‐up method from the medical history of 197 hereditary (C1‐INH‐HAE) and 20 acquired C1‐inhibitor deficiency (C1‐INH‐AAE), 3 factor XII and 3 plasminogen gene mutation (FXII‐HAE, PLG‐HAE) patients treated at our center between 1990 and 2020. The UAE group included edemas localized to the mesopharynx, hypopharynx, and larynx, as narrowing of these anatomical regions can lead to suffocation.

**Results:**

98/197 C1‐INH‐HAE (47 families) and 13/20 C1‐INH‐AAE, 1/3 PLG‐HAE, 1/3 FXII‐HAE patients had experienced UAE at least once according to their medical history. In case of C1‐INH‐HAE patients, in 6/47 families who had undiagnosed ancestors had 13 members who died of suffocation. After the diagnosis, 1‐1 member of two families died of UAE. 44/64 C1‐INH‐HAE patients did not smoke, 20/64 did. The occurrence of UAE was significantly higher in smoker patients. We analyzed 7607 HAE attacks of 56/98 patients. Out of all attacks, the incidence of UAE in the C1‐INH‐HAE group was 4%, and 9.5% in the C1‐INH‐AAE group, respectively.

**Conclusion:**

Early diagnosis is key in bradykinin‐mediated angioedemas cases, since the patient must be provided with adequate treatment; and also it is essential to inform patients about the importance of avoiding the trigger factors and the early symptoms of UAE, as these measures could significantly decrease the incidence of lethal UAEs.

**Abbreviations**
ACEI‐AAE: acquired angioedema related to angiotensinconverting enzyme inhibitors
AE: angioedema
ANGPT1‐HAE: hereditary angioedema with angiopoietin 1 gene mutation
C1‐INH: C1‐inhibitor
C1‐INH‐AAE: acquired angioedema with C1‐inhibitory deficiency
C1‐INH‐HAE: hereditary angioedema with C1‐inhibitor deficiency
FXII‐HAE: hereditary angioedema with factor XII gene mutation
HAE: hereditary angioedema
HS3ST6‐HAE: heparan sulfate‐glucosamine 3‐O‐sulfotransferase 6 mutation
InH‐AAE: idiopathic nonhistaminergic acquired angioedema
KNG1‐HAE: hereditary angioedema with kininogen 1 gene mutation
MYOF‐HAE: hereditary angioedema with myoferlin gene mutation
nC1‐INH‐HAE: hereditary angioedema with normal C1‐inhibitor
PLG‐HAE: hereditary angioedema with plasminogen gene mutation
UA: upper airway
UAE: upper airway angioedema
U‐HAE: hereditary angioedema of unknown origin

## 1 INTRODUCTION

The upper respiratory tract is the anatomical structure connecting the nostrils and the lips with the trachea, at which any pathological change can cause a stenosis. In clinical practice, however, upper airway (UA) stenoses are classified as emergencies that risk suffocation that can affect parts of the mesopharynx, hypopharynx, and the larynx. Classical symptoms of UA obstruction include lump sensation, dysphagia, hoarseness, aphonia, tachypnea, dyspnea, and stridor. One of the causes of UA stenosis can be airway obstruction caused by the edema. The upper airway edema (UAE) can be caused by inflammation due to UA infections, mechanical/chemical trauma of the larynx/pharynx, malignant tumors, or they can occur independently as well. In the last case, fluid from the intravascular space flows into the extracellular space (caused by vasoactive mediators); this process leads to the development of a local edema which can occur in the subcutaneous regions or the mucosa of the UA or the gastrointestinal tract.^1^ These types of angioedema (AE) can be divided into two groups. The first one is mediated by histamine/mast cells, which are often accompanied by urticaria and can be treated well with antihistamines. The other group is the group of bradykinin‐mediated AEs that are not accompanied by wheals and that do not react to conventional treatment (antihistamines [even in fourfold increased dose, which can be considered clinical diagnostic test in practice], glucocorticosteroids, adrenaline).^2^, ^3^, ^4^

Bradykinin‐mediated AEs have two types: hereditary and acquired ones. Hereditary angioedemas (HAE) can be divided into two groups: C1‐inhibitor (C1‐INH) deficient (C1‐INH‐HAE) ones and those where the level of C1‐INH is normal or slightly deficient (nC1‐INH‐HAE). C1‐INH deficiency can be proved with a complete complement laboratory testing (total classic complement cascade, C3, C4, C1‐INH concentration level and C1‐INH functional activity, anti‐C1‐INH antibodies [IgA, M, G]).^5^, ^6^, ^7^, ^8^ C1‐INH‐HAE is caused by the autosomal dominantly inherited mutation of the C1‐INH *SERPING1* gene, which results in the activation of the contact‐kinin‐kallikrein system, and which subsequently causes the liberation of bradykinin. Two types can be differentiated: the more frequent C1‐INH‐HAE type I (85% of cases), where the serum concentration level of C1‐INH is decreased; and the less frequent C1‐INH type II (15% of cases), where the antigenic level of C1‐INH is normal, but its functional activity is decreased. Approximately 25% of HAE patients have a negative family history, since the disease is developed due to a de novo mutation.^5^ Currently, seven forms of HAEs can be distinguished in the nC1‐INH‐HAE group: hereditary angioedema with angiopoietin 1 gene mutation (ANGPT1‐HAE); hereditary angioedema with factor XII gene mutation (FXII‐HAE); hereditary angioedema with kininogen 1 gene mutation (KNG1‐HAE), hereditary angioedema with plasminogen gene mutation (PLG‐HAE), hereditary angioedema with myoferlin gene mutation (MYOF‐HAE), hereditary angioedema with heparan sulfate‐glucosamine 3‐O‐sulfotransferase 6 mutation (HS3ST6‐HAE), and hereditary angioedema of unknown origin (U‐HAE).^9^, ^10^, ^11^, ^12^, ^13^, ^14^ There are acquired types of bradykinin‐mediated angioedemas as well, for example, acquired angioedema with C1‐inhibitor deficiency (C1‐INH‐AAE), acquired angioedema related to angiotensin‐converting enzyme inhibitors (ACEI‐AAE), and idiopathic nonhistaminergic acquired angioedema (InH‐AAE). In case of C1‐INH‐AAE, the classical pathway of the complement system is activated and the C1‐INH consumption is increased, which can be attributed to lymphoproliferative, tumorous, autoimmune, or infectious diseases, but autoantibodies against C1‐INH may also lead to inadequate C1‐INH function.^15^ The disease occurs later than the hereditary form and the family history is negative to AE.

AE is a rare side effect of ACEI therapy, which could occur either separately or as a provoking factor of C1‐INH deficient forms. In the Caucasian population, the incidence of AE in patients taking ACEI is approximately between 0.1% and 0.7%, but it is more common in Black people.^16^, ^17^ For 30% of patients presenting with angioedematous symptoms at the emergency care department, the possibility of the provoking role of ACEIs arises.^18^ For establishing this diagnosis, C1‐INH deficiency must be excluded.^16^, ^19^, ^20^ UAE is only 1%–2% of all AEs occurring in C1‐INH deficiency, but its significance resides in the fact that without adequate treatment it can lead to suffocation; UAE is the cause of the high mortality (approx. 30%) of C1‐INH‐HAE. Several retrospective studies examining C1‐INH‐HAE patients found that 40%–56% of the ancestors who showed the symptoms of the disease but were not diagnosed died of suffocation.^21^, ^22^, ^23^ Cicardi et al. set up the HAE triage system, which classification is based on the relationship of AE attacks of different localizations to respiratory distress. The location and extent of swelling, the duration of symptoms, and the rate of progression should be considered when assessing AE.^24^ Bradykinin‐mediated forms of AE generally develop more slowly than other AEs, but UAE can show a fulminant course.^25^

The purpose of the study was to analyze the clinical expression of UA angioedematous attacks in HAE and C1‐INH‐AAE patients treated in our center during the long‐term follow‐up.

## 2 METHODS

Our survey was performed with a retrospective long‐term follow‐up method using the medical history of 197 C1‐INH‐HAE (88 men, 109 women), 3 FXII‐HAE and 3 PLG‐HAE, and 20 C1‐INH‐AAE patients treated at the Hungarian Angioedema Center of Reference and Excellence (HACRE) between 1990 and 2020 (Table 1). The diagnosis was established based on family history, medical history, physical examination, complete complement laboratory testing (total classic complement cascade, C3, C4, C1‐INH concentration level and C1‐INH functional activity, anti‐C1‐INH antibodies [IgA, M, G]), and in case of nC1‐INH‐HAE patients, genetic testing.

**TABLE 1:** Demographic characteristics of patients with different angioedema

	All patients (number)			Patients, who experience upper airway edema (UAE) (number)		
	Total	Male	Female	Total	Male	Female
C1‐INH‐HAE	197	88	109	98	42	56
C1‐INH‐AAE	20	9	11	13	7	6
FXII‐HAE	3	1	2	1	0	1
PLG‐HAE	3	2	1	1	0	1

Abbreviations: C1‐INH‐AAE, acquired angioedema with C1‐inhibitor deficiency; C1‐INH‐HAE, hereditary angioedema with C1‐inhibitor deficiency; FXII‐HAE, hereditary angioedema with factor XII gene mutation; PLG‐HAE, hereditary angioedema with plasminogen gene mutation; UAE, upper airway edema.

Based on the family history, untreated ancestors of C1‐INH‐HAE patients were classified into the group with “suffocation due to UAE outcome” if the family member died of a suddenly developed suffocation, which could not be explained by any other underlying disease.

The number and type of AE attack for diagnosed patients was based on the data in the patient diary, hospital discharge reports, and medical records. The diagnosis of UAE was established if the laryngeal physical examination proved that the AE is localized to the area of the pharynx and larynx and/or the patient reported hoarseness, lump sensation, or difficulty swallowing or breathing. The lips and the tongue AE were not classified as UAE, since the edema in these areas does not cause airway obstruction without affecting the area of the mesopharynx.

All patients diagnosed with C1‐INH deficiency were trained about the clinical symptoms, with special focus on the recognition of the early symptoms of UAE and about the individual variability of the course of the symptoms. Each patient was supplied with the treatment for acute AE attacks: plasma‐derived (Berinert, CSL Behring GmbH) or a recombinant C1‐inhibiror concentrate (Ruconest, Pharming Healthcare, Inc). Moreover, icatibant (Firazyr, Takeda) was made available for all hereditary and acquired C1‐INH deficient, PLG‐HAE and XII‐HAE patients. For already diagnosed patients, family tree research and family screening were performed.

We analyzed the incidence, clinical properties, and the role of provoking factors. The connection between smoking and UAE was determined with Fisher exact test, with the significance level being *p* < 0.05. We used Mann–Whitney's *U*‐test, and considered *p* < 0.05 statistically significant for comparing baseline (at the time of diagnosis) C1‐INH functional level and the incidence of UAE. Data of AE symptoms and complement parameters were recorded in the National Angioedema Register.

The study protocol was approved by the institutional review board of Semmelweis University of Budapest, and informed consent was obtained from the participants in accordance with the Declaration of Helsinki.

## 3 RESULTS

### 3.1 Incidence of UAE in C1‐INH‐HAE patients

#### 3.1.1 UAE and suffocation in family history

156/197 patients were treated at HACRE who and/or whose family member(s) have experienced UAE; these patients were from 47 families.

Out of 156 patients, 32 (from six families) had 13 relatives who were not diagnosed and died of suffocation.

Relatives of 41/197 patients (from 20 families) treated at the Center did not have UAE; in this group, 2 members from 1 family had 2 not diagnosed ancestors who died of suffocation.

**FIGURE 1:**
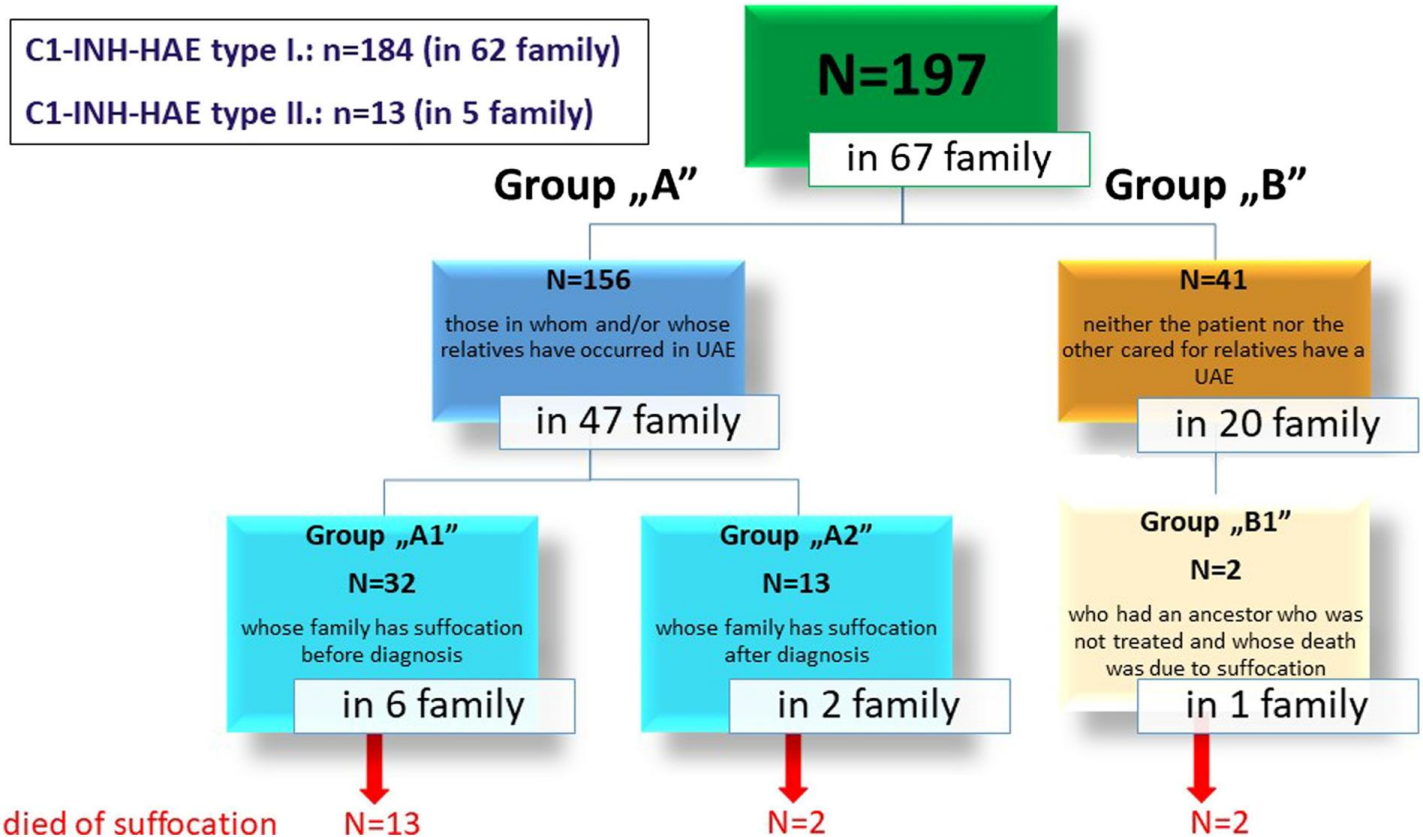
Death due to airway obstruction in C1‐INH‐HAE patients and their relatives. Group A gathers those patients who and/or whose families member(s) experienced upper airway edemas (UAE). Patients in group “B” their families members have never experienced UAE. After we analyzed the incidence of deaths due by suffocation in the families of both groups, which partially included those patients who were not treated at the Center. In group “A1”, 13 patients; in group “B1”, 2 patients; and in group “A2” another 2 patients died of suffocation. C1‐INH‐HAE, hereditary angioedema with C1‐inhibitor deficiency

After C1‐INH‐HAE diagnosis, we had 2 patients who died of UAE (1 male and 1 female, 23 and 37 years old, respectively) out of 13 patients from two families, due to inadequate compliance. The human plasma derived C1‐esterase inhibitor concentrate on‐demand treatment was available for both patients, but they did not use it. For one of the patients, it was her first UAE; this patient had C1‐INH‐HAE type II (Figure 1).

#### 3.1.2 Appearance of UAE in C1‐INH‐HAE patients treated at the HACRE

Out of the 29,017 HAE attacks of the C1‐INH‐HAE patients treated at the Center, 239 (0.8%) appeared on the lips and the tongue, 463 (1.6%) on the face, 975 (3.4%) appeared on the UAs, 987 (3.4%) appeared on the genitalia, 9288 (32%) on the gastrointestinal tract, and 17,065 (58.8%) attacks appeared in the subcutis.

During the follow‐up period, there were 98 patients (56 women, 42 men) who experienced an UAE during their lifetime. Out of those 98 patients, 90 patients had C1‐INH‐HAE type I while 8 patients had C1‐INH‐HAE type II. The median of the occurrence of the first UAE was at the age of 22 years (min: 3 years, max: 64 years). There was no difference between the sexes regarding the incidence of UAE: it occurred in 56/111 women and 42/86 men (*p* = 0.6672).

Out of 98 patients, the first UAE occurred after the diagnosis of C1‐INH‐HAE in 58, while in 40 patients, it occurred prior to the diagnosis. In the latter group, the median time between the first UAE and the diagnosis of C1‐INH‐HAE was 11 years (min: in 1 year, max: 54 years). At the time of the appearance of the UAE, this group either did not get treatment, or they received conventional (adrenaline, steroid, antihistamine) therapy. Four patients (all men) had tracheotomy, one patient had tracheotomy four times, because he had not responded to conventional treatment. Only 10/98 patients had UAE as their first angioedematous attack.

In case of 8/56 women and 7/42 men, the first UAE was provoked with a dental procedure or an operation in the mouth or the pharynx, and in case of one patient, it was caused by taking ACEI prior to the establishment of the C1‐INH‐HAE diagnosis.

#### 3.1.3 Association between UAE and functional levels of C1‐INH and the types of *SERPING1* gene mutations

**FIGURE 2:**
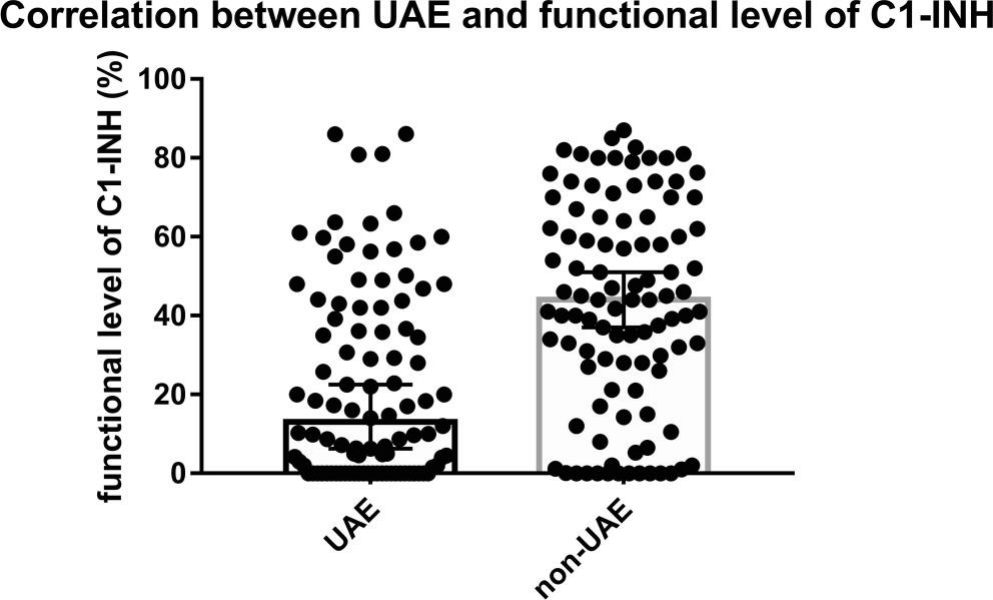
Correlation between upper airway edemas (UAE) and the baseline functional C1‐INH levels patients in the UAE group had significantly lower C1‐INH functional levels than those who had no UAE (*p* < 0.0001). The figure shows the median and 95% CI

We examined the level of C1‐INH functional activity, during which we found a significant difference between the UAE (median C1‐INH functional activity 13%) and non‐UAE (median: 44%) groups (*p* < 0.0001) (Figure 2). We also examined the relationship between the genetic types of the *SERPING1* gene and UAE. We had genetic results in 195 patients. Nonsense mutation in the *SERPING1* gene was confirmed in 16/98 (16.3%) cases among patients experiencing UAE, while in the group without UAE we found this type of mutation in 9/97 (9.3%) patients. The splice mutation was confirmed in 9/98 (9.2%) and 22/97 (22.7%) patients, respectively. The missense mutation was confirmed in 29/98 (29.6%) and 22/97 (22.7%).

#### 3.1.4 Smoking and UAE

From 2012, we have regular information about the smoking habits of our patients, so we were able to analyze 68/98 patients regarding their smoking habits. Four patients often changed their habits so they were excluded from the analysis. Forty‐four out of 64 patients were non‐smokers, while 20 smoked regularly. Examining the relationship between gender and smoking, we found no significant difference (*p* = 0.1607).

Out of the 44 non‐smoking patients, 50% only had UAE once during the examined period, while for smoking patients, this rate was 15% (*n* = 3), 75% experienced UAE more than once.

**FIGURE 3:**
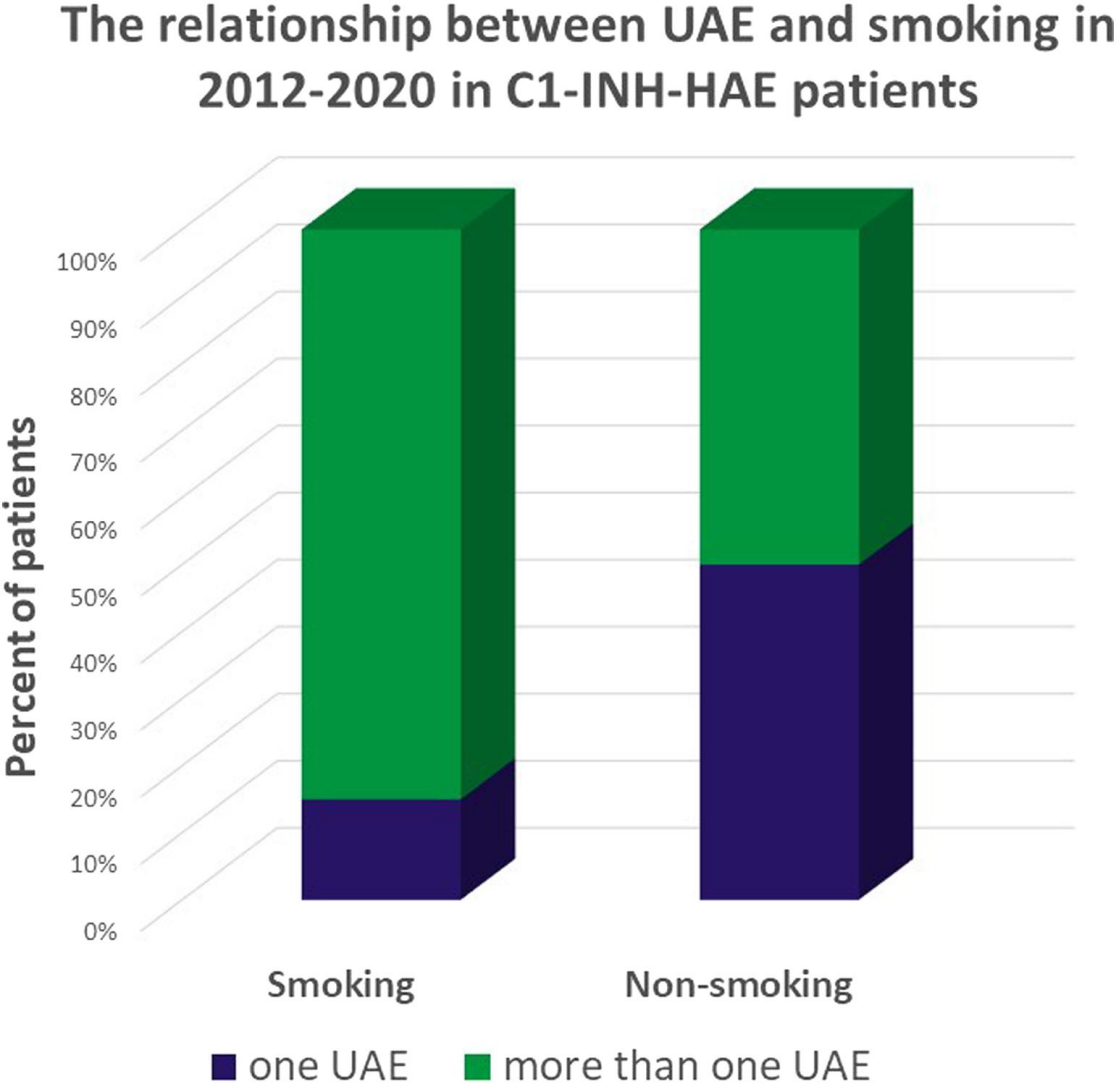
Incidence of smoking and the frequency of upper airway edematous attacks. Out of 44 non‐smoker patients, 22 experienced upper airway edemas (UAE) only once during the examined period (2012–2020). Out of 20 smoking patients, only 3 experienced one attack, 17 patients had more than one UAE

UAE attacks were more frequent in smokers, which was statistically significant. The Fisher exact test statistic value is 0.0119. The result is significant at *p* < 0.05 (Figure 3).

#### 3.1.5 C1‐INH‐HAE patients followed up between 2010 and 2020

**FIGURE 4:**
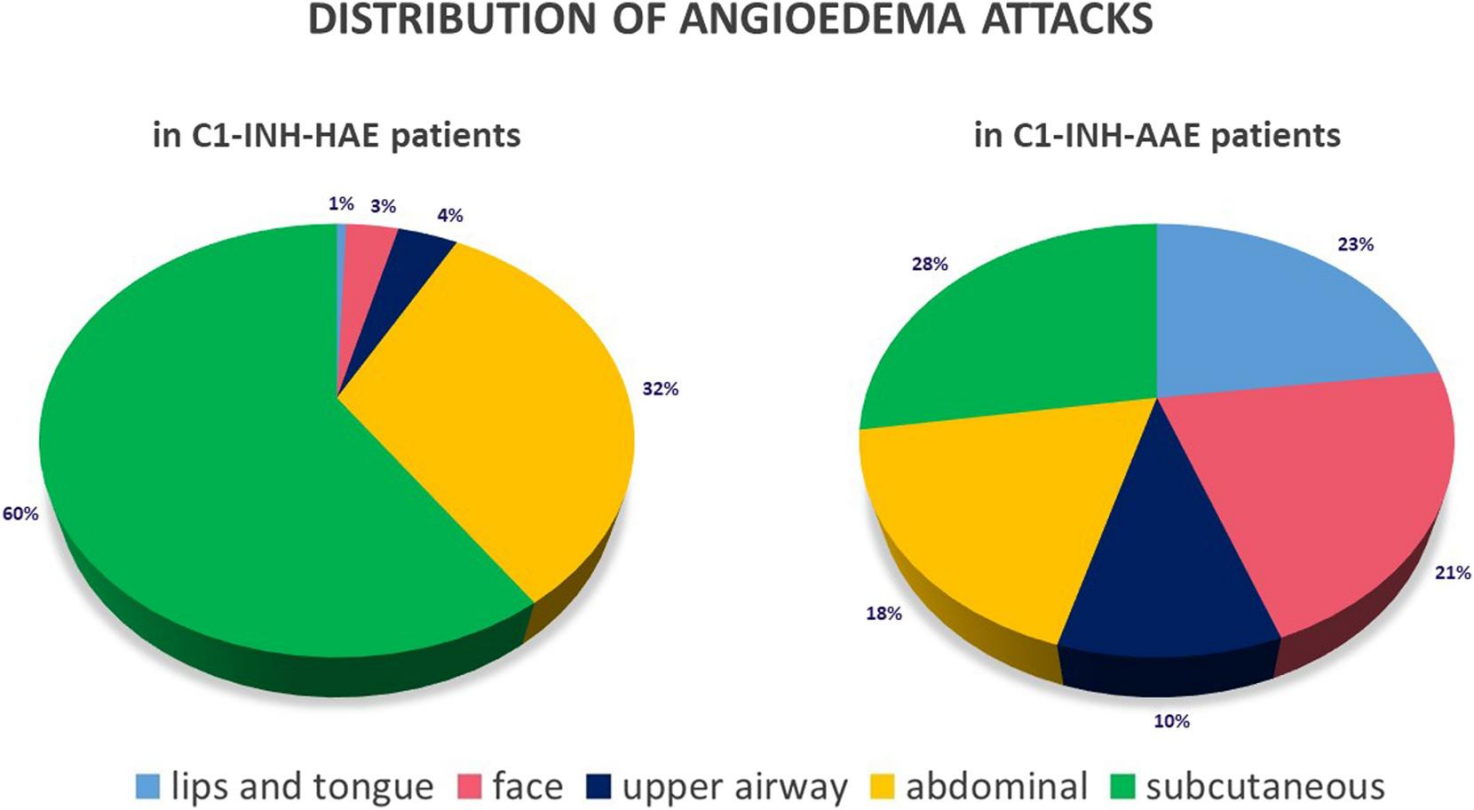
Distribution of the localization of angioedematous attacks in C1‐INH‐HAE and C1‐INH‐AAE patients. Between 2010 and 2020, 7607 attacks of 56 C1‐INH‐HAE patients were analyzed. Four percent of them experienced upper airway edemas (UAE). The number of all attacks was analyzed in case of 20 C1‐INH‐AAE patients due to the small patient number. UAE occurred in 10%. C1‐INH‐AAE, acquired angioedema with C1‐inhibitor deficiency; C1‐INH‐HAE, hereditary angioedema with C1‐inhibitor deficiency

We wanted to get a clear picture about the distribution of the attacks, so those patients were included in the group whose 2010–2020 data was available for the complete 10 years. We analyzed the data of 56 patients and 7607 attacks of these patients: 47 lips and tongue (0.6%), 262 face (3.4%), 301 UAE (4%), 2464 abdominal (32.4%), and 4533 subcutaneous attacks (60%) took place (Figure 4).

From the 301 UAE attacks, provoking factors were identified for 61 attacks. 16/301 attacks (for 14 patients) resulted from upper respiratory infections. Mechanical trauma (vocal stress, consumption of hot/cold food or drinks) could be named in case of 11/301 attacks (in seven patients). The UAE attack was provoked by dental procedures in four cases (in four patients), while menstruation provoked six attacks in four patients. 23/301 UAE attacks (14 patients) were provoked by stress and physical exhaustion. ACEI provoked an UAE in 1 patient.

We examined the frequency of the appearance of UAE attacks: 16/56 patients (28.6%) had one UAE attack in 10 years; 32/56 patients (57.1%) had 2–10 attacks; 4 patients (7.1%) had 11–20 attacks, and 4 patients (7.1%) had experienced more than 20 UAE attacks.

In case of 149/301 UAE attacks, our patients accurately documented when the attack happened. Based on this, four spikes can be observed in the frequency of UAE attacks: in January, April, July, and November.

**FIGURE 5:**
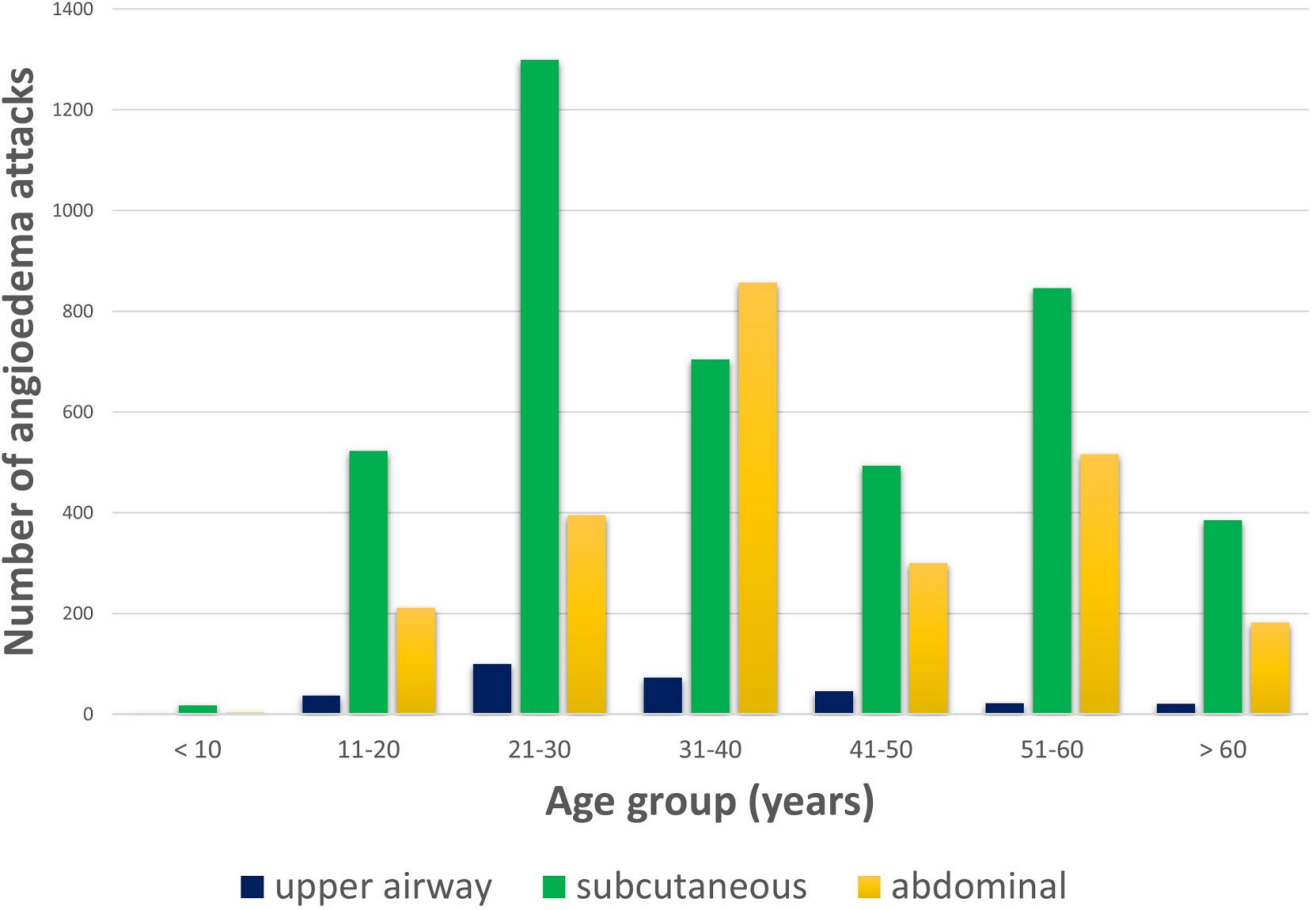
Age distribution of upper airway, subcutaneous and abdominal C1‐INH‐HAE attacks. Between 2010 and 2020, 7607 attacks of 56 C1‐INH‐HAE patients were analyzed. Subcutaneous attacks mainly occurred in the 21–30 and the 51–60 age groups; abdominal attacks occurred in the 31–40 and the 51–60 age groups, while upper airway angioedema was most frequent in 21–40‐year‐old patients. C1‐INH‐HAE, hereditary angioedema with C1‐inhibitor deficiency

The patients were divided into 10‐year‐long age groups and the localization of the attacks was also compared (Figure 5). The 4533 subcutaneous attacks mostly occurred in the 21–30 and 51‐60 age group; abdominal attacks occurred in the 31–40 and the 51–60 age groups, while in case of UAE attacks, there was a frequency spike in the 21–30 age group.

The age distribution of UAE showed the following distribution: 1 patient (2 attacks) between 9 and 10 years, 9 patients (37attacks) between 11 and 20 years, 17 patients (100 attacks) between 21 and 30 years, 11 patients (73 attacks) between 31 and 40 years, 15 patients (46 attacks) between 41 and 50 years, 7 patients (22 attacks) between 51 and 60 years, and 7 patients (21 attacks) at 60+ years.

### 3.2 Incidence of UAE in case of C1‐INH‐AAE patients

Out of 20 C1‐INH‐AAE patients treated at the HACRE, 13 have already experienced UAE. The family history regarding angioedematous attacks was negative for all patients. The median of the appearance of the first UAE was at the age of 60 years (min: 41 years, max: 83 years). In 5/13 patients, the first AE attack was localized as an UAE. For the first UAE, all 13 patients received conventional treatment. 3/13 patients (2 males, 1 female) were either intubated or had tracheotomy. In case of 8/13 patients, ACEI could be observed as a provoking factor. Out of 20 patients, ACEI provoked an AE attack in case of nine patients. One patient experienced UAE after a dental procedure.

10/13 patients had their C1‐INH‐AAE diagnosis established within 1 year after the first UAE. In case of three patients, the time between the first UAE and the diagnosis was 2, 7 and 10 years. In case of the first patient, chronic lymphocytic leukemia was the underlying disease, while the latter two patients have not had an underlying disease diagnosed since. Due to the small number of patients, all the data of C1‐INH‐AAE patients was analyzed focusing on the localization of the attack. The 20 patients had 187 subcutaneous (27.4%), 157 lip and tongue (23%), 146 face (21.4%), 124 abdominal (18.2%) and 69 UAE (10.1%) attacks altogether (Figure 4).

### 3.3 Incidence of UAE in case of hereditary angioedema patients with normal C1‐INH levels

In our center, three patients (from two families, one female, two males) are treated due to the mutation of the K330E plasminogen gene. From the 3 PLG‐HAE patients, two experienced AE: one patient at the age of 53 years had a tongue edema, the other patient had a face edema at the age of 17 and multiple tongue and pharynx edemas at the age of 40 years, which were probably provoked by taking ACEI (perindopril). We also treated three patients (from one family, two women, one man) due to the 18 base pair duplication in the 9th exon of factor XII (c894_911dup). Out of them, only one patient had AE: they experienced an attack that affected their face, eyelid, lip, pharynx, and abdomen at the age of 35. None of the patients smoked, and we do not know about anyone in their families who died of suffocation.

## 4 DISCUSSION

Currently, the first appearance and the frequency of UAE cannot be predicted, it occurred in all four examined patient groups (C1‐INH‐HAE, C1‐INH‐AAE, PLG‐HAE and FXII‐HAE), and it caused a life‐threatening condition and suffocation for the undiagnosed relatives of the patients in the C1‐INH‐HAE group, based on the family history. We examined nine families whose members have experienced suffocation due to UAE, and in case of 7/9 families, a total of 15 ancestors died of suffocation prior to the diagnosis of C1‐INH deficiency. This is in line with the findings of Bork et al., published in 2012, in which they pointed out the fact that death by suffocation is higher in undiagnosed patients. Almost 30% of the undiagnosed ancestors died of suffocation, and the rate of the death by suffocation in undiagnosed C1‐INH‐HAE patients compared to diagnosed patients was 9:1; the reason for this was presumed to be the lack of awareness in undiagnosed patients (the patient was not aware of the fact that UAE can occur anytime and that it can potentially be lethal).^26^ Although death by suffocation can occur even after the diagnosis, these are mostly caused by inappropriate treatment, which can result from the patients not getting targeted therapy, and from patients delaying or missing the administration of the available drugs, as it happened in case of two of our patients. In 2020, Perego et al. published their analysis, in which they examined the life expectancy and the cause of death in Italian C1‐INH‐HAE patients. They found that their life expectancy did not differ significantly from that of the general population; suffocation was preceded by death by malignant and cerebrovascular diseases, which could be explained by the availability of on‐demand treatment.^27^ The first UAE occurs at a young age, mostly in the second or third decade; however, it can also occur in children. In the literature the youngest age when UAE occurred was 3 years. UAE in children could rapidly cause suffocation since the diameter of the airways is way smaller than the airways of an adult; therefore a mild mucosa edema can cause an airway obstruction. In earlier studies, a connection was found between the early appearance of symptoms and the severity of the manifestation of HAE attacks.^28^, ^29^, ^30^, ^31^ Based on our follow‐up data, UAE attacks most often occur in the 21–30 age group, while subcutaneous and abdominal attacks frequently occur in the 51–60 age group. Earlier studies show that asphyxia is most common amongst patients between 21 and 30, and although the two patients diagnosed with HAE were from this age group, suffocation could occur at any age.^26^

It was an interesting observation that although HAE attacks occur more frequently and in more severe forms in women, the incidence of UAE attacks showed no difference between the two sexes. We found a similar result among men and women who smoked.^32^

The incidence of UAE in our patients is substantially higher than in earlier studies, where the incidence of UAE was less than 1% of HAE attacks. UAE covers 4% of all attacks in C1‐INH‐HAE patients, while it makes up 10% of all the attacks in C1‐INH‐AAE patients.^31^

The UAE group had significantly lower baseline functional C1‐INH levels, which consistent with the observations of our previous study which demonstrated a significant correlation between the level of functional C1‐INH and disease severity.^33^

Interestingly, nonsense *SERPING1* mutations were detected in a considerable number of patients who ever suffered from UAE (16.3%), compared to those who never had an UAE attack (9.3%), which is in line with our previous observation, where missense *SERPING1* mutations were associated with a less severe disease course while the nonsense and splice mutations resulted in a more severe course.^34^ On the other hand, previously confirmed splice mutations in *SERPING1* occurred less frequently in patients suffering from UAE (9.2%) compared to those who never had an UAE (22.7%). Many trigger factors are known which could provoke UAE, for example, upper respiratory tract infections, operations in the mouth‐pharynx‐larynx area, dental procedures, any kind of mechanical trauma in this region (e.g. endotracheal narcosis), or taking certain medications (ACEI).^30^, ^35^, ^36^, ^37^ Our patients mostly reported upper respiratory tract infections, stress, and physical exhaustion as the causes of UAE.

Since the area of the larynx is highly exposed to pathogens and irritative agents, it is presumed that the larynx has an important immunological function in the airways; this is also supported by the mucosa‐associated lymphoid tissue specific to the larynx found on the laryngeal surface of the epiglottis, the false vocal cords.^38^, ^39^ In a study published in 2017, it was found that smoking people have an increased ratio of CD31 T‐cells, regulator T‐cells and total T‐cells (i.e., cellular ratio of immune tolerance), which plays a role in modulating the immune homeostasis of the larynx (increased ImmunoCRIT values in smokers implicate a role for this environmental exposure in modulating laryngeal immune homeostasis).^40^ They showed that the chemical agents in tobacco smoke help neutrophil granulocytes produce superoxides and reactive oxygens; the necrosis of the mucosa could lead to the edema of the distal airways.^41^ We think it is an important observation that smokers experience UAE attacks more frequently than non‐smokers, which raises the question if tobacco smoke could cause similar changes to the UA mucosa. In view of this, we drew the attention of patients to the high risk of smoking in the development of UAE.

It was an important observation that UAE attacks in C1‐INH deficient patients show a different pattern. The time elapsed between the first UAE attack and the diagnosis was longer in case of C1‐INH‐HAE patients.

10/13 patients had the C1‐INH‐AAE diagnosis established within 1 year after the first UAE, which correlates with the diagnostic window in literature.^42^ However, it could be observed that patients who experienced UAE had a C1‐INH‐AAE diagnosis earlier.^43^

In case of bradykinin‐mediated angioedemas, establishing an early diagnosis is fundamental, as UAE has a crucial role in both hereditary and acquired AEs, being the only edema localization that can be lethal without proper treatment.

For the first time, hereditary types usually occur in young adulthood, but they can occur at any age; hence, patients must be informed and trained, which does not only include the early recognition of the starting symptoms of UAE (dysphagia, sore, lump sensation, voice changes),^44^ but also has to include the importance of avoiding trigger factors (smoking, medications, especially ACEI and infections). Concomitantly with the diagnosis, adequate medication for the treatment of two acute attacks and its continuous availability should be provided for symptomatic and asymptomatic patients too, because, as we have shown, even the first UAE can lead to suffocation in our patient group as well.

## CONFLICT OF INTEREST

Zsuzsanna Balla has participated in clinical trials of CSL Behring, Pharming, Pharvaris, and Takeda. Beáta Visy has participated in clinical trials of Takeda. Lilian Varga has received travel grants from CSL Behring and Shire Human Genetic Therapies Inc. Henriette Farkas received research grants from CSL Behring, Takeda, and Pharming and served as an advisor for these companies and Kalvista and Biocryst, and has participated in clinical trials/registries for BioCryst, CSL Behring, Pharming, Kalvista, Pharvaris, and Takeda. The other authors have declared that no conflict of interest exists.

## CODE AVAILABILITY

GraphPad Prism 5.0 (GraphPad Software, San Diego, California, USA).

## Data Availability

The data that support the findings of this study are available from Semmelweis University, Hungarian Angioedema Center of Reference and Excellence.
